# An introduction to the basic elements of the caste system of India

**DOI:** 10.3389/fpsyg.2023.1210577

**Published:** 2023-12-21

**Authors:** Vina M. Goghari, Mavis Kusi

**Affiliations:** Department of Psychological Clinical Science, University of Toronto, Toronto, ON, Canada

**Keywords:** caste, class, South Asia, India, Dalit, justice

## Abstract

Oppression, systemic bias, and racism have unfortunately long been part of the human experience. This paper is a review of basic elements of the Indian caste system, understanding its impact on the daily lives of different caste members, the role of colonialism in perpetuating the caste system, the Indian reservation system for mitigating disadvantages created by the caste system, and how categorization and labels can affect individual identity. This paper then discusses the global relevance of the caste system and its impact on mental health and psychological functioning. In India, the caste system is a comprehensive, systematized, and institutionalized form of oppression of members of the lower castes, particularly the Dalits. Formalized during the British colonial period, the caste system brings together two related Indian concepts of *varna* and *jāti* to create four social orders and multiple subunits. Sitting outside the traditional four orders are the Dalits, who experience social, economic, and religious discrimination due to an inherited status related to traditionally polluting occupations. Since the caste system extends beyond India to other South Asian countries, as well as to communities around the world that are home to the Indian diaspora, the inequities created by the caste system are a global issue. India’s affirmative action system provides important insights to policy makers, as well as researchers in the social sciences for how to counteract the effects of systematized oppression. Collectively, this can aid in a better understanding of the effects of discrimination and oppression on identity, self-esteem, and mental health, and how we can develop more targeted policies and procedures in our own local contexts.

## Introduction

There have been many re-ignited calls for anti-oppression, anti-racism, and justice in society, with the emergence of the #MeToo movement, Black Lives Matter, and Every Child Matters. However, these calls are not new. A related need for genuine reconciliation between diverse groups has echoed throughout our history across the globe. One group seeking to overcome historical and current discrimination are the Dalits within the caste system of India, a notable case given its long duration, its intersection with colonialism, and the connections between local religion, social norms, and economics. At the time of Indian independence in 1947, caste discrimination was recognized as one of the most pressing issues of this free and democratic nation, and the government instituted processes of reconciliation and justice. Today, given the international reach of the South Asian diaspora, the issue of caste is increasingly being recognized as a global issue. The history of the Indian caste system and the country’s strategies for justice and reconciliation are of interest in themselves, but can also provide many lessons for other nations. A study of the caste system can provide lessons for understanding psychological impacts on identity as an effect of marginalization.

## An overview of the Indian caste system

The Indian caste system is one of the oldest systematized and institutionalized forms of oppression, having been in existence for over 3,000 years (Thapa et al., 2021). At the bottom of this hierarchical system are groups such as the Dalits who experience caste-based discrimination, which impacts their access to health care, education, employment, and other social determinants of health (Thapa et al., 2021). To understand caste-based discrimination, it is important to understand the complexity of the caste system, the “book-view” and “field-view” perspectives of caste, and the role of colonialism in shaping the caste system as it exists today.

### The complexity of the caste system

The word “caste” is derived from the Spanish and Portuguese word *casta*, meaning lineage, breed, or race, and *casto,* meaning pure and unmixed (Oxford English Dictionary, 2023). In the 15^th^ century, Portuguese seafarers used the term for the first time in the Indian context, when they arrived to trade (Sahoo, 2017). Two Indian concepts frequently equated with caste are *varna* and *jāti*, though neither word captures the full structure currently known as the caste system.

The concept of *varna* – a term sometimes used interchangeably with caste-translates to “colour,” although within this historical context, it refers primarily to order and classification (Beteille, 1996). The hierarchical classification encompassed by *varna* entails a more flexible system of movement between four specific orders based on a person’s intrinsic nature (*svabhāv*) and qualities (*guna*) (Kumar, 2018). In the system clearly delineated within Hindu sacred texts, there can be only four *varnas* – that is, Brahmin, Kshatriya, Vaishya, and Shudra – since the *varnas* collectively represent discrete parts of the first being, Purusha. The Brahmins are the priests and teachers and were born from the head of Purusha; the Kshatriyas, the warriors and rulers, were born from the shoulders; the Vaisyas, or traders and merchants, were born from the thighs; and the Sudras, labourers and craftspeople, were born from the feet. The first three *varnas* were considered twice-born and hence purer than the fourth category, the Sudras, who were once-born. An unofficial fifth category, *avarnas*, existed outside the *varna* categories and were historically referred to by many names, including *achooots, Harijans,* or “untouchables.” Individuals from this lowest stratum of castes were considered to be impure and polluting since their inherited occupations often involved tasks considered to be physically and ritually polluting, such as working with dead bodies and animals or removing human waste (Thorat and Joshi, 2015). Dalit, meaning “broken” or “scattered,” is the term commonly used now to refer to this lowest of castes; the Indian government uses the term Scheduled Castes to refer to this group. The Adivasis, the Indigenous peoples, also known as Scheduled Tribes, also existed outside the four *varnas* and, like the Dalits, are the focus of affirmative action in India.

Second, the concept of *jāti* is more closely linked to the typical western concept of caste, originating as it does from the word *jana* (birth) and to the social identity ascribed by birth (Beteille, 1996). While *varna* is more about the system as a whole, *jāti* is more about the units, such as castes and communities, and is flexible in the sense that more categories can be created to meet societal needs (Beteille, 1996). Within each *varna,* there are many *jāti*s forming a complex hierarchy of occupational communities (Sahoo, 2017); consequently, it is estimated that 3,000 castes and 25,000 subcastes exist in India (Kumar, 2018). The Dalits comprise different castes based on various occupations and have their own hierarchy, as do the various other orders of the four *varnas*.

Defining the functions of caste is difficult given local variations. Acknowledging this, Ghurye (1969) delineated six defining characteristics of caste: (1) a society segmented into a system of groups that are predetermined at birth; (2) the system is hierarchical, although the hierarchy is often disputed; (3) the system restricts social interactions between upper and lower castes, such as eating together; (4) different castes are segregated, with lower castes living on the periphery of town with restricted access to resources such as wells; (5) occupations are generally inherited; and (6) endogamy (marriage within one’s own caste) prevails, although hypergamy (marriage into a higher caste for women) is permitted. However, formal definitions of caste have been criticized in view of regional variations in practice and the flexible nature of day-to-day interrelations between castes. As B. R. Ambedkar, a Dalit activist who chaired the committee to draft the constitution of India, stated: “Caste is not a physical object like a wall of bricks or a line of barbed wire which prevents the Hindus from co-mingling and which has, therefore, to be pulled down. Caste is a notion; it is a state of the mind” (Ambedkar, 2014). As Ambedkar highlights, to bring true integration, policies need to change the culture, and thereby the mindset of people.

### Balancing our understanding of caste through multiple sources of evidence

In understanding the caste system, it is important to recognize that a distinction exists between the “book-view” understanding of *varna* and *jāti* from Brahmin-authored sacred Hindu texts, such as the Vedas and Manusmriti, and the real-life practice of the caste system, which one can learn from regularly speaking with and observing members of different castes (Sahoo, 2017). From the book-view of Hinduism, scholars surmise that caste is a religious and cultural construct – a social practice of the Indian subcontinent and distinct from practices within Western societies. For example, focusing on Hindu sacred texts can promote an overemphasis on purity/impurity dimensions of the caste system. The “field-view” perspective, on the other hand, focuses on how caste is practiced in everyday life (Sahoo, 2017). Studies of caste through local traditions and customs suggest that, in addition to or instead of traditional notions, caste is also shaped by numerical strength, economic and political power, and even Western education and occupations (Sahoo, 2017). For example, Srinivas (1984) discusses how the nuances of caste play out by discussing the limitations and permutations of power within each caste. He notes that the Kshatriya had power, while Brahmins, though powerful themselves, had to subjugate themselves to kings and perform any rituals the kings desired. Furthermore, to solidify their role, Brahmins had to make themselves essential to kings through knowledge of law, ethics, and religion. Srinivas (1984) also points out that notions of impurity sometimes transcended caste divisions: not only are Dalits viewed as impure, but Brahmins who performed funeral rites were sometimes also referred to as “untouchables” – degraded and made impure by that work despite being Brahmin.

The concept of Sanskritization, which was introduced by Srinivas (1956), also exemplifies how the nuances of caste play out in everyday life. Sanskritization refers to the process through which caste upward mobility was made possible; albeit, upward mobility through Sanskritization was more possible for the middle than lower castes. In practice, Sanskritization was the process of taking on aspects of the Brahminic way of life (e.g., adopting vegetarianism and abstinence from alcohol), as well as adopting Brahminic rituals and beliefs (e.g., concepts of karma, dharma). It is noteworthy, that such strategies are not guaranteed to work, and if the strategies do work, it typically takes one to two generations before the change in status is accepted by other castes. Unfortunately, Dalit groups are unable to transcend their “untouchability” even through Sanskritization; though that did not stop that process in these groups (Srinivas, 1956).

Some scholars have argued that the Dalits accepted their societal role because they believed that their position would improve through reincarnation into their next life. However, anthropological fieldwork conducted in India demonstrates that these concepts of rebirth (samsara), karma, and dharma were rarely used by individuals from lower castes, who might not have understood their meaning (Deliege, 1993). For instance, Kathleen Gough reported that when she asked a group of older Pallars, a Dalit community, about concepts of death, duty, destiny, and rebirth, they apparently replied:

“Mother, we don’t know! Do you know? Have you been there?”

I said, “No, but Brahmans say that if people do their duty well in this life, their souls will be born next time in a higher caste.”

“Brahmans say!” scoffed another elder. “Brahmans say anything. Their heads go round and round”. (Gough, 1973, p. 234)

Analysis of origin myths within Dalit communities shows a high degree of consistency in ideology. While caste is portrayed as a given, Dalit origin myths do not frame caste as strictly hierarchical, unchangeable, or necessarily imposed by God (Deliege, 1993). Myths contain elements that explain lower caste status as resulting from misunderstanding or trickery. Deliege (1993) recounts an example:

In the beginning, there were two brothers who were poor. Then they went together to pray to God. God asked them to remove the carcass of a dead cow. The elder brother answered: “Een thambi pappaan” (My younger brother will do it) but understood: “Een thambi paappaan” (My younger brother is a Brahman); since that very day, the younger brother became Brahman (paappaan) and the elder brother became a Paraiyar. All castes originate from these two brothers. (p. 536)

Hence, based on a misunderstanding the older brother in this myth becomes the Paraiyar, while the younger brother becomes the Brahmin. Since by tradition, older brothers are considered heads of households after their father and stand hierarchically above their younger brothers, the reversal of fortune in this myth arguably promotes the subversive view that the Paraiyar’s status exceed that of the Brahmin.

Deliege (1993) notes that such origin myths are difficult to uncover and are not part of the core identity of Dalits today as they are more concerned with social, political and economic issues than with ritual purity. Given such nuances, it is important to use caution in accepting analyses of caste based strictly on Hindu and/or Brahmanical scriptures or texts, including academic work. Overarching statements about the caste system typically fail to capture the complexities of caste-related local practices, the views of Dalits, customs and necessities, or their evolution over time.

### The role of colonialism in perpetuating caste

Although the caste system is typically viewed as intrinsic to India and presented in contrast to Western customs, it is important to acknowledge the role of European colonialism in cementing the caste system. Under British rule in the Indian subcontinent (the British Raj), “caste” became a way to organize and systematize India’s diverse forms of social identities and communities, and was used for all government executive actions (Dirks, 1992, 2001). Technological innovations spearheaded by British colonizers – such as roads, railways, telegraph, newspaper and mail systems, and printing – enabled castes to organize themselves in new ways through travel and correspondence. For example, printing allowed castes to print and formally record their constitutions rather than leave them in malleable form (Srinivas, 1957). Riser-Kositsky (2009) writes that “colonial policies, through their structuring and politicization of caste, were one of the direct causes for the incessant and often deadly caste conflicts in India today” (p. 31).

Moreover, it is problematic to use Western conventions as a baseline by which to judge the “correctness” of Indian conventions or behavior, and apply it with broad strokes. Many conceptions of the Indian caste system originate from colonial British perceptions of the structure. For instance, Riser-Kositsky (2009) quotes senior British officials who, from the beginning, viewed caste as a problematic institution that allegedly embodied “indolence, avarice, lack of cleanliness, venality, and ignorance” (p. 31) Furthermore, the British officials viewed the caste system as emblematic of the moral inferiority of the Indian character compared to their own, given its supposed “lax hold of facts, its indifference to action, its absorption in dreams, its exaggerated reverence for tradition, its passion for endless division” (pp. 35–36). Hence, views of the caste system were used to justify negative views of Indians compared to the British.

Despite their views of the caste system, the British officials made minimal effort to dismantle the system directly; indeed, they tied caste intricately to Hinduism, thereby framing caste as an insurmountable convention (Riser-Kositsky, 2009). Instead of dismantling the caste system, the British tried to appease and accommodate the lower castes by digging separate wells, setting up special schools, and starting reservation policies. Although this had positive benefits for lower castes, it did not overhaul the system. Moreover, many scholars (see for review, Riser-Kositsky, 2009) cite the British census efforts to capture caste categorization as creating more rigid caste identities and promoting caste identity over other social identities. New incentives such as scholarships or military recruitment motivated various groups to have their caste classified in a particular way. Riser-Kositsky quotes Ambedkar, who in 1943 acknowledged the entrenchment of caste in Indian institutions and day-to-day life, writing that “today the census is a matter of first rate concern to everyone, as Indian politics devolved into a numbers game in which every side tried its best to cook the books” (p. 42).

It is therefore important not to base notions of the Indian caste system solely on portrayals by British colonials, given their lack of holistic understanding of Indian regional identities. Furthermore, even positive British accomplishments such as technological implementations, may have also had unintended negative consequences in solidifying caste identities and inter-caste conflict in ways that continue today. Finally, continuing to define caste as a solely Hindu construct impedes progress for other South Asian religious groups as well as for Dalit Hindus who have converted to different religions to escape caste discrimination, but continue to face it regardless.

## The relevance of studying the Indian caste system

The caste system is a global issue as it exists across communities in the growing South Asian diaspora (Jodhka and Shah, 2010). Recently, caste-based discrimination has been recognized as an issue in North American city councils, companies, and academic institutions, with some of these institutions implementing measures to prevent caste-based discrimination (Dave, 2022; Venkatraman, 2022; Singh, 2023a,b). As a system that categorizes and stratifies individuals, caste influences self-identity and has adverse effects on self-esteem and mental health (Jaspal, 2011; Komanapalli and Rao, 2021). It is important to understand how caste features and functions across communities in the South Asian diaspora given the global reach of caste, the problem of caste-based discrimination in schools, workplaces, and other institutions, and the impact of caste on identity and psychological functioning. Moreover, understanding the Indian affirmative action system, especially for policy makers and other groups that are advocating for equity, diversity, and inclusion in their local contexts, could generate new ideas.

### Affirmative action in practice: reservation system

The Indian Constitution outlined three primary strategies to reduce caste discrimination: (1) Legal and regulatory measures that introduced penalties for caste based discrimination (e.g., The Untouchability (Offences) Act, 1955; The Employment of Manual Scavengers and Construction of Dry Latrines (Prohibition) Act, 1993); (2) Allocation of resource and benefits to develop measures to reduce the socio-economic gap between Scheduled Castes and higher castes and (3) compensatory discrimination, including a reservation system for public and government institutions (Saxena, 2004).

India has the oldest affirmative action program in the world, launched in 1950 just a few years after India’s independence from colonial rule. India’s reservation programs, which is largely a quota-based system, is referred to by many terms, including affirmative action, positive discrimination or compensatory discrimination. Quota systems existed in a different domain during the British Raj, including reserved electoral seats for different religious groups as well as lower castes (Saxena, 2004). In independent India, the compensatory discrimination program focused at first on Scheduled Castes and Scheduled Tribes in the public and government sector, namely education, employment, and legislative bodies. At first these provisions were supposed to be in place for only 10 years; however, they are still deemed a necessity with no sign of dissipating (Haq and Ojha, 2010). In the 1980s, “Other Backward Classes” (i.e., communities that were identified as below the national average on social or educational indicators; Ramaiah, 1992) were added to the program. In 2019, “Economically Weaker Section” (EWS) of society was also added to the program (Pillalamarri, 2022). This category included individuals of higher castes who needed social and economic assistance, as other groups already had provisions in the Constitution.

In 1992, the Supreme Court of India ruled that reservation quotas could not exceed 50 percent of total seats. Anything above 50 percent, the Court judged would violate equal access as guaranteed by the Constitution, thus they placed a cap on reservations (Pillalamarri, 2022). However, in 2022, the Court ruled to uphold the 10% reservation for the EWS, even though the constitutional amendment to include these provisions meant that up to 59.5% of seats in central institutions could be reserved for disadvantaged groups. Justice Jamshed Burjor Pardiwala wrote in his verdict that:

“Reservation is not an end but a means—a means to secure social and economic justice. Reservation should not be allowed to become a vested interest. Real solution, however, lies in eliminating the causes that have led to the social, educational, and economic backwardness of the weaker sections of the community”. (Pillalamarri, 2022)

One of the criticisms of the reservation system is that benefits accrue to the most economically and socially advantaged of the disadvantaged groups. The term “creamy layer” was first coined by Justice Krishna Iyer in the case of the State of Kerala vs. NM Thomas, wherein he observed that “benefits of the reservation shall be snatched away by the top creamy layer of the backward class, thus leaving the weakest among the weak and leaving the fortunate layers to consume the whole cake” (Gupta and Giri, 2015). To mitigate this issue, in 1992, the Supreme Court defined “creamy layer” and what constituted “backwardness.” For reservations for the Other Backward Classes, certain restrictions were put into place to take advantage of the reservation system: those for whom reservations applied could not be (1) children of high officials as per the constitution, (2) children of civil servants in high positions, (3) children of high-ranked armed-forces officials, (4) children of professionals and those engaged in trade and industry, (5) children of property owners, and (6) children of people with an annual income exceeding 8 lakh (National Commission for Backward Classes, 1993). This was to prevent the economically, socially, and educationally advantaged members of the Other Backward Classes from reaping all the rewards from the reservation system.

Overall, reservations for Scheduled Caste communities within public sector employment, as well as in higher education have helped individuals from communities that would have previously been unable to access or benefit from these spheres (Haq and Ojha, 2010). In central government services, the percentage of employees from the Scheduled Castes increased from 11.31% to 16.18% from 1977 to 1987 and many of the gains were for professional and managerial jobs (10.23% to 18.63%) (Haq and Ojha, 2010). In the public sector enterprises from 1977 to 1980, the number of Scheduled Castes increased from 7.42% to 17.44% (Haq and Ojha, 2010). In higher education institutions from late 1970s to late 1990s, the number of Scheduled Castes increased from 7% to 7.8% (Haq and Ojha, 2010). However, in all sectors, the data also showed that reserved seats continued to go unfilled.

Helping the most disadvantaged and rural communities remains a further goal, and programs to improve elementary and high school education to better prepare candidates for higher education are also warranted (Haq and Ojha, 2010). Similarly, making workplaces more inclusive will make the experience of work better for individuals from underrepresented communities and facilitate integration (Haq and Ojha, 2010). Deshpande and Yadav (2006) outline how India’s reservation policies could be re-formulated to target those most in need. The authors outline the many merits of the quota system, including creating political solidarity and loyalty, being comparatively easy to administer, and being generally robust to misuse. As Deshpande and Yadav (2006) very eloquently state:

“Of course, all affirmative action is inherently contentious because it seeks to alter the status quo of inter-group power equations. Issues like this form the very stuff of politics, and better policy design alone will not make these contestations vanish. On the other hand, bad policy design will certainly make things much worse, because it will ensure that political costs are much higher than they need to be and that the social benefits are either too meagre, or badly targeted, or both. Thus, apart from its primary objective of enabling the attainment of social objectives (such as equality of opportunity or elimination of unjust inequalities), policy design also has the important responsibility of ensuring efficiency in the sense of minimising unavoidable costs and maximising potential benefits.” (p. 2419)

Deshpande and Yadav (2006) argue the second point has not received sufficient attention. The authors suggest four principles be included in the design of such affirmative action policies: (1) be evidence-based; (2) incorporate multiple dimensions of disadvantaged status, in addition to caste; (3) be attentive to the intersectional effects of the different status dimensions; and (4) be attentive to the severity of disadvantage. By including these principles in policy, Deshpande and Yadav (2006), stress not only will greater social justice be achieved, but the sole focus on caste as identity will be dissuaded. By focusing on indicators that are related to disadvantaged identities, rather than the identity itself, it breaks down the focus on caste. Also, by now focusing on measurable indicators of disadvantage, it allows for those indicators to be debated, measured, and studied; hence, progress for different disadvantaged groups can be monitored and changes can be made in a non-contentious way. This will also allow the extremely contentious politicization of caste quotas, especially for Other Backward Classes, to be reduced, as progress can easily be monitored of included groups, as well as the inclusion of new groups as necessary. This will bring transparency and accountability to decision-making, which will help reduce caste politics.

### Necessity and adverse reification of categorization and labels

One problem arising from emphasizing caste as the main mechanism of identity is that caste may become more central to identity than other underrecognized and intersectional factors. Hence, measures taken to promote economic and social equity within caste systems may themselves perpetuate the system, since caste identity is already a determinant of economic outcomes and social standing (Srinivas, 1957). One factor cited in promoting caste is that caste has a political dimension, and hence politicians who want to abolish caste also vie for votes from these different electorates (Srinivas, 1957).

Also, given the politicization of caste and that benefits through the reservation system are largely based on caste classification, some castes have sought to be re-classified as lower castes. For instance, some groups in the Other Backward Classes category have wanted to be reclassified as Scheduled Castes or Tribes, with one prominent example being the Gujjars in Rajasthan seeking to be reclassified as a Scheduled Tribe (Many die as Indian caste demands lower status, 2008; Goswami, 2019). Similarly, the Jats in Haryana, the Patels in Gujarat, and the Marathas in Maharashtra, three groups that are prominently involved in agriculture, have held prominent demonstrations, at times violent, to be re-classified as Other Backward Classes to access benefits of the reservation system. This push to be reclassified as lower castes has occurred despite these groups being closer to the dominant groups in social and economic indices than the Other Backward Classes, Scheduled Castes, or Scheduled Tribes (Deshpande and Ramachandran, 2017). Surprisingly, the Jats and Marathas did receive legislative support to be re-classified as lower castes; however, this was later overturned by different courts to protect resources for the most disadvantaged groups (Deshpande and Ramachandran, 2017). This process of seeking downward classification is in contrast to the traditional process of Sanskritization to seek upward mobility, and a result of economic unrest and competition for quotas in India’s affirmative action system. For India, it will be quite the task to have strategies to deal with legitimate economic and social concerns of more dominant classes, while maintaining protection for disadvantaged groups.

A humorous parable which circulated in the Times of India (Deshpande, 2007) highlights one view of how identity in the caste system works in practice. In the scenario, India sends a 20-member space exploration team to the moon. The caste quotas for the crew are decided immediately: six SC [Scheduled Castes], four ST [Scheduled Tribes], eight OBCs [Other Backward Classes], and last but not least – if possible – two astronauts. The joke surmises that one can learn the castes of the “astronauts” and that the most vital piece in describing them is their qualifications and occupation – not their caste. However, members of the other categories are referred to with their caste as the focal point despite also being qualified astronauts. In this way, members of upper castes are entitled to an explicit identity that exists outside of their caste, whereas the identity of members of the lower castes is always tied to their caste first and foremost in the public sphere. As Satish Deshpande eloquently writes in his article that highlights this parable:

This, then, is the predicament of caste today: its invisibility – or persistent denial – in one context versus its hypervisibility – or constant invocation – in another. India is split into two irreconcilable parts. One part appears to be divesting itself of caste, having climbed on to a plateau of economic and educational security where the normal rules of the game are now in its favour. But the larger part of society is still heavily invested in caste, because it is trying to climb the steep slope of inherited disadvantage, and caste is the only lever it has to reduce the tilt of the playing field. These unequal and opposed parts are also mutually reinforcing in a strange way. It is as if each must weave what the other must unravel.

Deshpande (2007) concludes that in order to annihilate caste, it must be formally acknowledged and spoken of, and that members of the upper caste must lead the way in doing so because they wield the most power in the public sphere.

Beteille (1996) echoes similar sentiments that while caste continues to provide security and attachment in a chaotic world, the continuation of the system is not a natural inevitability. He agrees with Srinivas that caste ultimately would evolve more into a form of ethnicity. Beteille (1996) also argues that the reason the caste system has continued is due to the failures of the Indian government to fulfill its promises of equal access in education, health care, and other public institutions, which would help to develop necessary connections between different communities. Hence, caste identity has become more entrenched as people rely on economic support from members of their own communities, which consists of people with shared identities such as caste, religion, and the village or district where they were born in India. However, as we discuss in the next section, the caste system exists on a global scale, not just in India. Therefore, its continued existence cannot in current times be due solely or primarily to the failures of the Indian government in providing equal access to public institutions and economic opportunities.

The process of self-identification and group formation is complex, and by singling out caste identity as the core factor to use primarily for categorization for affirmative action in India, although unintentional, has further solidified caste identity as legitimate; thus, making it more difficult to eradicate. Given that caste is categorization based on division of a traditional labour system, it is difficult to see the value in retaining this unfair arbitrary system; hence, the ultimate goal of the Indian government has been its eradication. However, over time, caste identity has come to represent a shared communal and cultural background. Moreover, similar to other marginalized groups, different Dalit groups are re-absorbing their own identities in a process of re-appropriation and pride in their resiliency and history. Hence, it does not seem that caste as an identity is anywhere close to eradication, and perhaps the goal should first shift to eradicating caste-based discrimination.

### Caste discrimination as a global issue

One compelling reason for social scientists and other academics outside of South Asia to learn about the caste system, is that it is a global issue. Although traditionally discussed and treated as an issue specific to India and Hinduism, caste also exists in other historically and geographically related South Asian countries (Jodhka and Shah, 2010). Jodhka and Shah (2010) have noted that although variations exist between South Asian countries, caste-based forms of social and economic discrimination are present in all these countries. Nepal, a majority Hindu country, openly recognizes caste discrimination and, like India, has a National Dalit Commission to identify Dalit castes and ameliorate caste-based discrimination. By contrast, Sri Lanka does not recognize a caste system, although it does exist in a relatively less rigid form and is practiced differently by the country’s three main ethnic groups: Sri Lankan Tamils, Indian Tamils, and Buddhist Sinhalas. Similarly, both Bangladesh and Pakistan have scheduled castes that apply only to their Hindu populations, although caste discrimination is also found among both country’s Muslim and other religious populations. Particularly in Pakistan, for example, the small religious minority of Christians who largely converted from Hinduism, did not lose their traditional Dalit status and face intersectional discrimination (Jodhka and Shah, 2010).

Acknowledging the global reach of caste, Dr. B. R. Ambedkar noted that “if Hindus migrate to other regions on earth, Indian caste would become a world problem.” Accordingly, North American countries have begun to include caste as a protected identity alongside gender and racial identity, sexual orientation, etc. Caste discrimination in North America first came to public attention through the technology industry. For instance, the California State Court will hear a case in which a Dalit ex-employee of Cisco claims that he was a victim of caste discrimination by his supervisors who allegedly denied him promotion opportunities and excluded him from meetings (Moreno and Smith, 2022). The claimant also alleges that Cisco retaliated against him for complaining about how he was being treated. The claim was originally filed in 2020. In that same year, Apple added a prohibition of caste discrimination to its conduct policy (Dave, 2022). Earlier in 2023, the city of Seattle, USA, became the first city to outlaw caste discrimination (Singh, 2023a). The public school board in Toronto, Canada, has also recently recognized that caste discrimination operates within the school system and has requested that the Ontario Human Rights Commission create a framework to address the issue (Singh, 2023b). Similar to other academic institutions such as Harvard, the California State University system has added caste as a protected identity across its 23 campuses (Venkatraman, 2022). These examples acknowledge the widespread experience of caste discrimination across communities in the South Asian diaspora.

To study this issue, a Dalit civil rights organization called the Equality Labs^1^ administered a web-based questionnaire to members of the South Asian diaspora in the United States – that is, individuals originating from Bangladesh, Bhutan, India, Nepal, Pakistan, Sri Lanka, the Maldives, Trinidad/Tobago, Guyana, Fiji, Tanzania, and Kenya (Zwick-Maitreyi et al., 2018). Results of the survey, which was open to people of different religious, political, tribal, and caste affiliations, demonstrated a picture of caste discrimination in North America. Twenty-five percent of Dalits who were surveyed reported that they faced verbal or physical assault based on caste; 33% reported experiencing discrimination during their education; 66% reported being treated unfairly at their workplace; 60% experienced caste-based derogatory jokes or comments; 40% were made to feel unwelcome at places of worship; 20% felt discriminated against in a business; over 40% felt rejected in romantic relationships due to caste; and 50% lived in fear of their caste status being “outed” (Zwick-Maitreyi et al., 2018). This data clearly demonstrates that caste discrimination is not restricted to India or even to South Asia, it affects the diaspora in North America and likely beyond; therefore, not only do more studies need to be done on caste discrimination in North America, but it is important for people to be aware of the system. Indeed, this awareness is only a first step, as parents, supervisors, peers, social acquaintances, etc., might be dealing with issues of caste discrimination in the workplace or in their social circles.

### The impact of caste status on psychological functioning

A greater number of studies that investigate the impact of caste status on psychological functioning are necessary. Existing studies have generally found people who are classified as members of lower castes to have poorer mental health (e.g., self-reported feelings of depression and anxiety) compared to people who are classified as higher caste members. For instance, in a population-level (*n* = 960) study of depression and help-seeking amongst people in the Uttarakhand district in north India, Mathias et al. (2015) found higher rates of depression among people who were classified as Scheduled Castes or Scheduled Tribes compared to those who were classified as members of higher castes. Gupta and Coffey (2020) found similar results in a population-level analysis of the effects of caste-based discrimination on the mental health of people who were classified as Scheduled Castes in India, using data from the World Health Organization’s Survey of Global Aging and Adult Health (WHO SAGE). Specifically, Gupta and Coffey (2020) found that respondents who were classified as Scheduled Caste members were more likely to report being mildly, moderately, severely, or extremely depressed in the last month. These individuals were also more likely to report experiencing anxiety in the last month. In contrast, the respondents who were classified as higher caste members were more likely to report having good mental health. When Gupta and Coffey (2020) controlled for differences in socioeconomic status (i.e., educational attainment and asset wealth such as owning a car, a motorcycle, a refrigerator, or livestock) between the respondents who were classified as Scheduled Caste and higher caste members, they found that while the gap in reported feelings of depression could be explained by differences in assets and educational attainment, the differences in reported feelings of anxiety still remained. This indicates that socioeconomic disparities might not entirely account for differences in mental health outcomes. Caste has an impact on mental health even when controlling for differences in socioeconomic factors. It should also be noted that discrimination is often an underlying cause of differences in socioeconomic status (Williams, 1999; Gupta and Coffey, 2020). Hence, caste-based discrimination can indirectly impact mental health outcomes by leading to disparities in economic and educational attainment.

Discrimination has long been identified as a risk factor for mental illness (see for review, Williams, 2018; Vargas et al., 2020). As it relates to caste, studies point to caste-based stigma, social exclusion, violence, and unfair treatment as factors that contribute to poor mental health as well as suicides in Dalits (Jaspal, 2011; Komanapalli and Rao, 2021). In biographies and ethnographies in which Dalits provide accounts of their experience of caste-based discrimination, the feelings that are often described include feelings of shame, inferiority, exclusion, isolation/loneliness, and servility to those who are considered as higher caste members (Paik, 2014; Gidla, 2017). Jaspal (2011) reports that the perception that one’s in-group is stigmatized or devalued by society can have a negative impact on the self-esteem of those who are members of that group. This is particularly likely as individuals are often seen as “interchangeable exemplars” of their in-groups. Hence, the caste system’s devaluation of people who are classified as Scheduled Castes threatens the self-esteem of individuals in those groups. The caste system stigmatizes and dehumanizes them as “untouchable,” and creates a climate where they can be judged and devalued for being members of those groups.

## Lessons and future directions

Our motivation for writing this paper was to inform social scientists about the global reach of the caste system and the need for more studies to illuminate how caste-based identity and discrimination features and functions not only in South Asia, but also in North America and other parts of the world. Given the long history and adaptation of the caste system over time as well as the attempts at reconciliation and justice, there are many lessons that are applicable to our attempts at equity, diversity and inclusion today. These lessons include:

1. Being cautious about basing knowledge on a complex issue solely on a “book view” rather than the day-to-day lives of individuals. In the case of India’s caste system, a sole focus on Brahaminical Hindu texts provides a one-sided view of caste, with an over emphasis on the purity and pollution dimension of the caste system. Field work demonstrates that Dalits have their own origin stories of caste that are different from the Brahmanical scriptures. While these origin stories may also serve to legitimize the system, they give a different view of caste by focusing on how being assigned a lower or “untouchable” caste was through error or treachery, rather than any fault or intrinsic nature of a caste. Therefore, it is important to understand the caste system from different points of views as well as how caste is implemented in daily life through the interactions of different castes. An extension of this is the need to understand how the caste system has changed with time.
2. Being cautious about basing knowledge of a system solely on colonial writings or sentiment as well as not recognizing the role of colonialism on the cultures of colonized nations. In the case of India’s caste system, viewing the system solely as a Hindu anomaly, neglects to recognize the role of the British Raj on taking a more informal system and concretizing it.
3. Learning from India’s process of trying to reduce systemic discrimination against the Dalits through policy that is largely based on quotas. While the reservation system has been helpful in addressing some of the socioeconomic disparities between Scheduled Castes, Scheduled Tribes and the higher castes, it has not completely closed the gaps in socioeconomic outcomes between these groups nor has it stopped caste-based discrimination or its impact on the mental health outcomes of Scheduled Castes and Scheduled Tribes. The reservation system also serves as a case study of how a redistribution system can have the unintended consequences of intensifying identities and perpetuating a system (i.e., the caste system) that should be eradicated.
4. Learning more about group identity formation and how it affects groups differentially in a complex hierarchical system over time. In the case of India’s caste system, over time, caste identification has become politicalized and impacts upper and lower castes differentially, with upper castes getting to become “caste-less” to some extent, whereas to accrue government and public benefits, lower castes are still defined by their caste. In addition, with caste identity now intricately tied to accrual of government and public benefits, upper castes seek to be reclassified as Scheduled Castes, Scheduled Tribes, or Otherwise Backward Classes, which is a change from the past when castes were pursuing upward mobility through Sanskritization.
5. Learning more about the globalization of discrimination and oppression through the experiences of the diaspora. In the case of the caste system, experiences of the South Asian diaspora and responses of the different communities in which they are embedded, provides an opportunity to better understand caste-based discrimination. It also highlights the need for more knowledge about how caste-based discrimination manifests in these global communities and how we might enact reconciliation in the international context. Moreover, the South Asian diaspora is heterogenous with recent immigrants, international students at Colleges and Universities, first- and second-generation individuals, people with more distant South Asian ancestry, and individuals with different caste identities. It would be helpful to know what caste discrimination looks like in these populations. Is it present in all the different subgroups and to what extent?
6. Research is also needed to understand the impact of caste on the psychological functioning of South Asians. Similar to other identity variables such as age, gender, race, and sexual orientation, a better understanding of caste status could further the understanding of psychological factors such as self-esteem, mental health, coping, etc., in South Asians. Moreover, understanding the interaction between caste and socioeconomic status and other identify variables, such as gender and age, would be beneficial.

## Conclusion

India’s caste system has captivated individuals both locally and globally as a divisive and highly discriminatory categorization system. At the bottom of the hierarchy are the Dalits, who face the most extreme discrimination, due to the traditional polluting occupations they were born into. Although the caste system is intrinsic to Hinduism with its strong link to content in Brahmanical scriptures, it is found in different religious and cultural groups in South Asia, and even Dalits, who convert to different religions, continue to be stigmatized and discriminated against due to their lower caste status. The caste system is changing with time, functioning more as ethnic groups. Categorization infiltrates all orders, and even Dalits have a hierarchy of castes and traditionally practiced social, economic and financial exclusion of lower castes within their own order. The caste system was inherently more flexible prior to colonialism and the process of labelling and categorization started in the British Raj has, in a double-edged way, intensified caste identities, which continues on today.

As caste is a system found beyond India, more needs to be done in recognizing the needs of Dalit communities in other South Asian countries and in the diaspora. Currently, in North America, there is more attention on caste-based discrimination, under the broader lens of equity, diversity, and inclusion. However, Dalits are finding that there are not enough outlets for complaints of bias as most equity, diversity, and inclusion policies in North America do not cover caste as a protected identity. Moreover, most North Americans are not knowledgeable about the caste system and how it is a global issue.

Often when thinking of issues of justice and reconciliation, we think predominantly within our local context and Euro-Western ideologies. Issues of anti-oppression and reconciliation are global issues and it can be helpful to learn lessons from other systems and ideologies. Given that the caste system of India is one of the oldest and all-encompassing forms of hierarchical discrimination, it is a system that we should study in terms of its function, features, and impact on those who have been classified, stratified, and devalued under the system. Indeed, many comparisons have been made regarding racial bias in the United States to the caste system of India, with Black Americans being classified at the bottom of the hierarchy and treated in a similar manner to the Dalits of India. One prominent example of this view is the book by Isabel Wilkerson (2021), *Caste: The Origins of Our Discontents*. In summary, the caste system of India, South Asia, and the world is an evolving entity, which provides many lessons of both individual and systematized discrimination, as well as lessons about reconciliation and justice at a global level.

## Data availability statement

The original contributions presented in the study are included in the article/supplementary material, further inquiries can be directed to the corresponding author.

## Author contributions

VG conceptualized and wrote the paper. All authors contributed to the article and approved the submitted version.

## Footnotes

^1^ https://www.equalitylabs.org/
